# An Estimate of the Total DNA in the Biosphere

**DOI:** 10.1371/journal.pbio.1002168

**Published:** 2015-06-11

**Authors:** Hanna K. E. Landenmark, Duncan H. Forgan, Charles S. Cockell

**Affiliations:** United Kingdom Centre for Astrobiology, School of Physics and Astronomy, University of Edinburgh, Edinburgh, United Kingdom

## Abstract

Modern whole-organism genome analysis, in combination with biomass estimates, allows us to estimate a lower bound on the total information content in the biosphere: 5.3 × 10^31^ (±3.6 × 10^31^) megabases (Mb) of DNA. Given conservative estimates regarding DNA transcription rates, this information content suggests biosphere processing speeds exceeding yottaNOPS values (10^24^ Nucleotide Operations Per Second). Although prokaryotes evolved at least 3 billion years before plants and animals, we find that the information content of prokaryotes is similar to plants and animals at the present day. This information-based approach offers a new way to quantify anthropogenic and natural processes in the biosphere and its information diversity over time.

## Abstract 2

The diversity of life on Earth is typically considered in terms of the total number of species. However, this essay, by estimating the total amount of DNA in the biosphere at 5.4 x 10^31^ megabases, offers an information-based view of biodiversity.

## An Information View of the Biosphere

**Fig 1:**
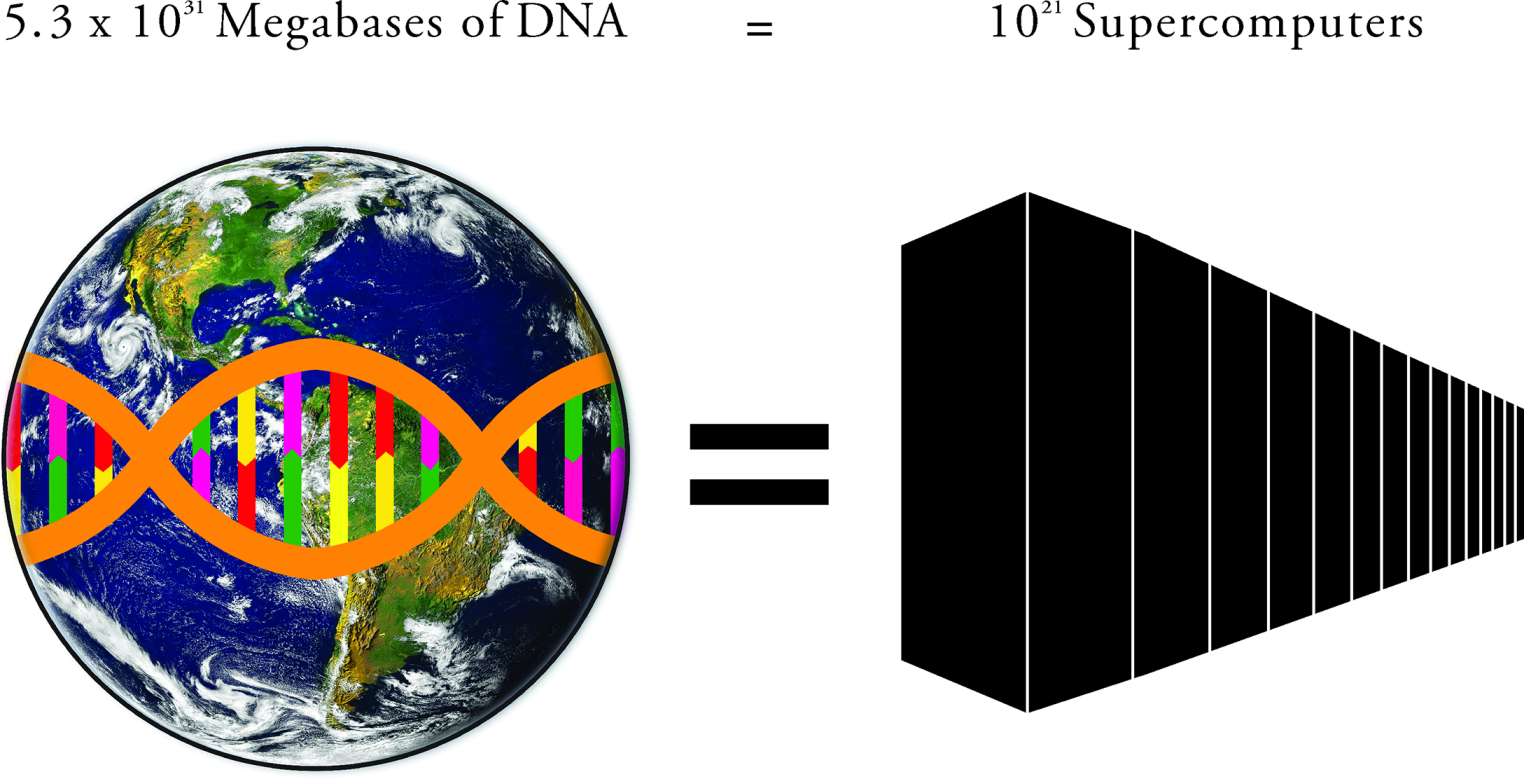
Storing the total amount of information encoded in DNA in the biosphere, 5.3 × 10^31^ megabases (Mb), would require approximately 10^21^ supercomputers with the average storage capacity of the world’s four most powerful supercomputers. Image credit: Globe from NASA, Wikimedia Commons; Composite Fig. 1 created by David Hammett.

Biodiversity and habitat loss is recognised as a global issue [1]. In response, substantial research effort has been invested in genome sequencing and the preservation of vulnerable species and habitats. However, despite these remarkable advances, to our knowledge, there is still no estimate of the total information content of the biosphere. Using available DNA sequencing and genome data, combined with large-scale surveys of biomass, we present an alternative way of quantifying and understanding biodiversity. This is accomplished by adopting an information view of biodiversity, in which the total amount of information in the biosphere is represented by the available amount of DNA (Fig 1). In this way, the biosphere can be visualised as a large, parallel supercomputer, with the information storage represented by the total amount of DNA and the processing power symbolised by transcription rates. In analogy with the Internet, all organisms on Earth are individual containers of information connected through interactions and biogeochemical cycles in a large, global, bottom-up network. By combining data on genome size, spatial diversity, and mass from different prokaryotes, eukaryotes, and the viruses, we estimate the total biomass for each group and then derive a first-order, lower-bound approximation for the total DNA content of each group.

This approach to evaluating the information content of the biosphere has implications in several fields of science. An important current priority is understanding diversity loss in the biosphere. The modern approach is to focus on species diversity [2]. However, species are merely the phenotypic representation, or containers, within which the information that underpins the functioning of the biosphere is stored as DNA. Counting the number of species and number of individuals as a measure of biological diversity yields limited insight into the quantity of the information that the biosphere contains. The corresponding action in the electronic computer analogy would be to attempt to estimate the information content of the Internet by counting the number of models and number of computers attached to it. Here, we instead use an approach whereby the total amount of DNA is quantified, giving an estimate of the information content in the biosphere.

We note that the approach that we propose here (and the analogy of supercomputers) does not necessarily imply a global, Gaia-like superorganism. We merely observe that ultimately all organisms interact with each other and the environment. Thus, the information being processed in the biosphere is interlinked in a large mass of organisms, however one chooses to conceptualise this. It does not have to be considered as a single, self-regulating organism. The manner in which the total information in the biosphere is processed, and the degree to which it is coordinated and interlinked in feedback processes, is another matter, but one that could be investigated using an information-based approach.

## The Total DNA in the Biosphere

Using information on the typical mass per cell for each domain and group and the genome size, we estimate the total amount of DNA in the biosphere to be 5.3 × 10^31^ (±3.6 × 10^31^) megabase pairs (Mb) (Table 1). This quantity corresponds to approximately 5 × 10^10^ tonnes of DNA, assuming that 978 Mb of DNA is equivalent to one picogram [3]. Assuming the commonly used density for DNA of 1.7 g/cm^3^, then this DNA is equivalent to the volume of approximately 1 billion standard (6.1 × 2.44 × 2.44 m) shipping containers. The DNA is incorporated within approximately 2 × 10^12^ tonnes of biomass and approximately 5 × 10^30^ living cells, the latter dominated by prokaryotes. By analogy, it would require 10^21^ computers with the mean storage capacity of the world’s four most powerful supercomputers (Tianhe-2, Titan, Sequoia, and K computer) to store this information [4]. The methodological approach is summarised in Box 1, and detail is provided in S1 Methods.

**Table 1:** The total DNA content in the biosphere

	DNA amount (Mb)
Prokaryotes	1.6 (1.1) × 10^31^
Unicellular eukaryotes	1.3 (0.9) × 10^29^
Fungi	1.7 (3.4) × 10^27^
Animals	4.2 (1.5) × 10^29^
Plants	3.6 (3.4) × 10^31^
Viruses	4.0 (3.4) × 10^29^
**Total**	**5.3 (3.6)** × **10** ^**31**^

**Box 1. Methods Summary**

To estimate the total information content of the biosphere, DNA was quantified in five major subgroups of life: prokaryotes, plants, animals, unicellular eukaryotes (sometimes referred to as protists), and fungi. For each group, available quantifications of biomass, number of individuals, or their respective densities were converted to DNA quantities through appropriate conversions (S1 Methods), including average genome size. For prokaryotes, the estimated total number of cells, 5 × 10^30^ cells [5] was combined with the average prokaryotic genome size, 3.2147 Mb [6], as determined by Pulsed Field Gel Electrophoresis (PFGE), to give the total amount of DNA contained in the group. For plants, the average biomass from four different estimates, 561.8 Gt of carbon [5], 520 Gt of carbon [7], 1,841 Gt biomass [8], and 890 Gt biomass [9], was converted to the number of cells, assuming carbon content is 50% of dry weight and using a plant cell mass of 2 × 10^–10^ g [10] and, lastly, converted to a total amount of DNA of 3.65 × 10^31^ Mb using an average genome size of 5,958.01 Mb [11]. DNA quantities in the animal kingdom were found using estimates for the total biomass in major subgroups of animals (S1 Methods), which was converted to a total number of cells using a human cell mass of 1 × 10^–9^ g [12]. For each group, the number of cells was combined with the average genome size for that group, taking the mean of the relevant available genome size entries in the Animal Genome Size Database [13], before the total DNA amount was summed from the individual contributions, to give a final DNA quantity in animals of 4.24 × 10^29^ Mb. An alternative approach was also employed, whereby animal biomass densities from different habitats and biomes were used to find a global animal biomass using biome data (S1 Methods), which combined with the average animal genome size of 4,456 Mb [13] resulted in an animal DNA content of 3.67 × 10^29^ Mb. The abundance of unicellular eukaryotes was based on density measurements of algae, ciliates, amoebae, and testacea from different biomes: Austria (meadow, beech forest, spruce forest) [14], Australia (arid) [15], Puerto Rico (rainforest) [16], Scotland (upland grassland) [17], United States (coniferous rain forest, desert) [18,19], and Bangladesh (water) [20]. Using the average genome size of 855.59 Mb [21] for algae and 59.529 Mb [22] for other unicellular eukaryotes, a DNA quantity in unicellular eukaryotes of 1.31 × 10^29^ Mb was established. Biomass densities above and below ground were used to estimate the total DNA content of fungi as 1.73 × 10^27^ Mb, using an average genome size of 31.874 Mb [23] and eukaryotic cell mass of 2 × 10^–10^ g [10]. Viruses also contribute to the total DNA available on Earth. The total number of viruses on Earth has been estimated at 10^31^ [24], which, combined with an average viral genome size of 0.039518 Mb [25], gives a DNA content in viruses of 3.95 × 10^29^ Mb. Other DNA that was not included in the estimate of the total DNA in the biosphere is chloroplast DNA (approximately 0.12–0.2 Mb), mitochondrial DNA (mtDNA, approximately 0.0165 Mb in humans), plasmids (approximately 0.001–1 Mb) and extracellular DNA in the environment. Owing to their small genome size compared to the nuclear genome size, they are unlikely to have an order-of-magnitude effect on the total DNA estimate we derive. Fossilised DNA is assumed not to be playing a role in the computational capacity of the biosphere. We did not take into account leaf litter, which has been estimated to have a biomass of 122 Gt [7]; assuming a plant genome size of 5,958 Mb, this gives a total DNA contained within litter of 7 × 10^30^ Mb. This material is analogous to old garbage data. The total DNA amount in the biosphere was, hence, found to be 5.3(3.6) × 10^31^ Mb. Uncertainties were quantified for all groups (S1 Methods).

The total information content of prokaryotes was found from the product of the mean genome size of prokaryotes and estimates of the total number of prokaryotes on the earth, giving a value of 1.6 × 10^31^ Mb. It was estimated to be similar to the total DNA in all eukaryotic groups, 3.7 × 10^31^ Mb. In the eukaryotes, the DNA was calculated to predominantly reside in plant matter. Four different estimates of global plant biomass, converted to DNA quantity, gave a total plant DNA content of 3.65 × 10^31^ Mb. We were able to calculate the total DNA in all animal groups using two methods. One method used the mass of different types of organisms and then extrapolated to total numbers of organisms, their total cell mass, and thus DNA content. The other method used specific biome biomass estimates to calculate the global DNA content in animals by considering the mass distribution across different biomes on Earth. Both methods gave final DNA quantities within 2-fold of each other (4.24 × 10^29^ and 3.67 × 10^29^ Mb, respectively). The former was used in our final estimate. Although, in our estimates, fungi and unicellular eukaryotes contribute less DNA than plant or animal matter, they still contain a substantial quantity of the processing power, having 1.73 × 10^27^ and 1.31 × 10^29^ Mb, respectively. We enumerated them using existing estimates for their biomass in different biomes on the earth. Although viruses are not cellular life, they play an enormously important role in biological interactions in the biosphere and the turnover of carbon, for instance [24]. They are, therefore, influential in the computational processing occurring in the biosphere. We estimate the total DNA contained within them (or their equivalent RNA code, which we include here because, unlike transcribed RNA in cellular life, the RNA in some viruses is used as their permanent genetic code) to be 3.95 × 10^29^ Mb.

Several orthogonal methods were employed to test the accuracy of the result. We utilised data on DNA concentrations in soil and water to achieve a much coarser-grained global estimate of the quantity of DNA in the environment. The quantity of dissolved DNA in aquatic environments is of the order of 10 μg/l [26] and the total volume of aquatic habitats on Earth is 1.4 × 10^21^ l [5,27], giving a total quantity of DNA of approximately 1.4 × 10^31^ Mb. Similarly, the concentration of DNA in soil is about 10 μg DNA/g soil [28,29]. The earth has about 1.1 × 10^21^ g soil, using a bulk density of 1.3 × 10^6^ g/m^3^ [5,30], hence the total quantity of DNA is estimated to be 1.1 × 10^31^ Mb. Thus, we estimate the amount of DNA in soil and water to be of the order of 10^31^ Mb. Information on DNA abundance can also be gleaned from the earth’s total organic carbon, which is estimated to be on the order of 2,000 Gt [31]. DNA makes up a few percent of cellular carbon; from literature estimates we chose a working value of 3% DNA in cellular organic carbon [32,33], giving a total DNA quantity of 5.9 × 10^31^ Mb. Although these orthogonal approaches target different parts of the biosphere and do not resolve groups of organisms in a similar way to the more detailed calculation that forms the core of this paper, the order-of-magnitude estimates they produce agree with our more detailed estimate, lending support to its accuracy and the size of the associated uncertainty.

## Assumptions in the Approach

In calculating the total amount of DNA, we are assuming that every base pair is a unique piece of information. One could also estimate the number of DNA base pairs in the total number of unique functional genes in the biosphere to calculate what minimal total amount of DNA would need to be stored to recreate all the transcribed genes on the earth. We view the calculation of the total number of base pairs as a first-order estimate of the information content of the biosphere to be the right approach for several reasons. All organisms uniquely interact with other organisms and their environment and, thus, viewed as an interconnected web of information, even two clonal organisms containing two sets of identical DNA contribute to the total information processing in the biosphere. For most organisms, there is a minimum viable population, below which they become functionally extinct. Therefore, individual organisms, even ones containing substantially the same genetic information, are contributing to the sustainability of the total information content of the biosphere. In the case of a genuinely redundant organism, its DNA is analogous to stored, back-up information in electronic computing.

Other DNA that was not included in the estimate of the total DNA in the biosphere is chloroplast DNA (approximately 0.12–0.2 Mb), mitochondrial DNA (mtDNA, approximately 0.0165 Mb in humans), plasmids (approximately 0.001–1 Mb) and extracellular DNA in the environment. Owing to the small size compared to the nuclear genome size, they are unlikely to have an order-of-magnitude effect on the total DNA estimate we derive. Fossilised DNA is assumed not to be playing a role in the computational capacity of the biosphere. We did not take into account leaf litter, which has been estimated to have a biomass of 122 Gt [7]; assuming a plant genome size of 5,958 Mb, this gives a total DNA contained within litter of 7 × 10^30^ Mb. This material is analogous to old garbage data.

The genome size data used here are derived from different C-value databases. Within each database, the C-values typically come from a variety of sources. The prokaryotic C-values came from a database based on PFGE values, giving a difference in the second significant figure compared to a database using sequenced values.

## The Computational Power of the Biosphere

Finding the amount of DNA in the biosphere enables an estimate of the computational speed of the biosphere, in terms of the number of bases transcribed per second, or Nucleotide Operations Per Second (NOPS), analogous to the Floating-point Operations Per Second (FLOPS) metric used in electronic computing. A typical speed of DNA transcription is 18–42 bases per second for RNA polymerase II to travel along chromatin templates [34] and elsewhere suggested as 100 bases per second [35]. Precisely how much of the DNA on Earth is being transcribed at any one time is unknown. The percentage of any given genome being transcribed at any given time depends on the reproductive and physiological state of organisms, and at the current time we cannot reliably estimate this for all life on Earth. If all the DNA in the biosphere was being transcribed at these reported rates, taking an estimated transcription rate of 30 bases per second, then the potential computational power of the biosphere would be approximately 10^15^ yottaNOPS (yotta = 10^24^), about 10^22^ times more processing power than the Tianhe-2 supercomputer [4], which has a processing power on the order of 10^5^ teraFLOPS (tera = 10^12^). It is estimated that at 37°C, about 25% of Open Reading Frames in *Escherichia coli* are being transcribed [36], but this is in a metabolically active population. In the natural environment, the percentage of DNA being transcribed is likely to be much less. Nevertheless, it is clear that even if the total DNA in the biosphere being transcribed at any given time was orders of magnitude less, the biosphere has many orders of magnitude more computational power than the fastest electronic computers yet built [4].

## DNA in the Biosphere through Time

An information-based view of the biosphere may provide a way to consider the changing complexity of the biosphere through time. For example, mass extinctions can be considered to be similar to physical hard drive damage in a computer. This analogy is particularly appropriate to the case of a hard shock caused by asteroid or comet impact, as proposed for the end-Cretaceous extinction [37]. Although statistics such as the destruction of 75% of life at the end-Cretaceous boundary are impressive, the true importance of this to the biosphere’s information content and processing power would depend on the genome sizes of extinct organisms, what influence the extinction had on microbial populations, and the effects on DNA transcription rates (altered by changed metabolic states in a stressed biosphere). In analogy to electronic computing, efforts have been made to recover lost information in “de-extinction” attempts, such as recovery of the Pyrenean ibex (*Capra pyrenaica pyrenaica*) [38]. These efforts to reconstruct software from destroyed hardware are still in their infancy.

Comparing the quantity of DNA in microorganisms, plants, and animals shows that there is a remarkable similarity in size of the contributions, within two orders of magnitude. This similarity is surprising, given that prokaryotes evolved at least 3 billion years before plants and animals. The likely reason for this is the larger genome size in eukaryotes. Although the cell numbers in eukaryotes are approximately two to five orders of magnitude lower than the number of prokaryotes (animals by biome: 4.11606 × 10^25^ cells; animals by biomass: 1.28907 × 10^26^ cells; plants: 2.44730 × 10^28^ cells, compared to prokaryotes: 5 × 10^30^ cells [see Methods]), the average genome size in prokaryotes is 3.2147 Mb, compared to the higher value in plants (5,958 Mb) and animals (4,456 Mb). The higher genome size in animals and plants may reflect the bioenergetic possibilities in eukaryotes in general that allow for more complex genetic machinery [39].

We might also wonder about the future computational power of the biosphere. In the next billion years or more, will the information content or the computational speed increase, and how will they be distributed amongst the major domains and subgroups of life? Eventually, when the sun’s luminosity increases sufficiently and the planet moves towards a greenhouse state, animal and plant computational contributions will be destroyed and prokaryote computational power will deteriorate as conditions on the planet become more extreme [40]. Ultimately, even the last vestiges of microbial life will diminish to a point at which their populations can no longer be sustained. At that point, the biosphere supercomputer will be destroyed.

## Uncertainties and Future Questions

Our estimate of the total DNA in the biosphere has a number of uncertainties. Hence, the calculations outlined here should be treated as a lower-bound estimate. The uncertainties tell us much about what knowledge we lack on the biosphere and so, as an exercise, calculating the total information content in the biosphere has the potential to reveal a great deal about our own knowledge. Although there is considerable data on the genome size distribution of different groups of organisms, we do not know the relative biomass of organisms with the different classes of genome size. We found a surprising lack of large-scale surveys of biomass densities in different biomes and across different groups, particularly for fungi and unicellular eukaryotes, and to a lesser extent for animals, plants, and prokaryotes. Uncertainties arise in converting biomass estimates into DNA quantity because of the lack of data on cell mass, which is almost absent from the literature, and also from uncertainties in biomass and C-values. Seasonal changes in the biomass of life are also poorly understood. Despite impressive advances in molecular biology and genome sequencing of species, our analysis emphasises that we still lack very fundamental data about the biomass of different types of life on Earth and their distribution, which is essential for refining estimates of the information content of the biosphere.

For all organisms, a ploidy level of 1 was assumed, as average ploidy levels across groups are poorly constrained. The exception to this is for animals, for which a ploidy level of 2 was used, as animals are virtually always diploid. C-values, by definition, refer to a haploid (monoploid) cell, but the nomenclature on this topic is somewhat ambiguous [41]. Treating all groups except animals as monoploid underscores the fact that our estimate is a lower bound. For plants and fungi, in which ploidy levels vary from one to a few during the life cycle, the effect is unlikely to have an order of magnitude effect. The fungal and plant genome databases used in this study gives an average fungal ploidy of 2.6 and an average plant ploidy of 2.9. Only for prokaryotes is a greater variation observed. Bacterial ploidy varies from monoploid to about 120 copies of the genome per cell in one known case with an apparent minority of bacterial species having true monoploidy. Most surveyed species of bacteria have a ploidy of less than ten, hence the effect on the total estimated quantity of DNA would be less than an order of magnitude [42]. The situation is much the same for archaea, with some haloarchaea shown to have between 10–25 copies of the genome, determined by growth stage [43]. We know very little about the distribution of ploidy levels in different biomes and in different organisms in the natural environment. Improving our understanding of the global environmental distribution of ploidy would go far to improve the quantification of the total DNA in the biosphere.

To advance our understanding of the processing power of the biosphere we need more detailed data on transcription rates in organisms in the natural environment. Most studies that examine transcription are laboratory-based. Although the reasons for this can be understood—laboratory organisms are more tractable and more amenable to the complex apparatus needed to measure transcription rates—a systematic study of transcription in the natural environment would yield much information not just about the processing power in the biosphere but also about the general physiological state and activity of genetic systems in the wild. This would allow us to make a more accurate assessment of the NOPS of the biosphere.

There are a number of other worthwhile calculations that could be undertaken. A calculation of the total quantity of DNA being replicated in the biosphere, coupled with error rates in different organisms, would give us a new quantitative way to measure the rates of production of variation in the biosphere—the raw material on which evolutionary selection pressures act. This would provide a way to quantify the process of evolution at the planetary scale. To accomplish this successfully, we need to know a great deal more about DNA replication rates in diverse organisms from prokaryotes to animals in different environments and biomes around the world and DNA error rates in the natural environment under different conditions. An estimate of the total DNA in the biosphere is the first step in that direction.

An estimate of the total number of amino acids being added to polypeptide chains per second would tell us something about biomass production in the biosphere as well as the metabolic activity in cells. To accomplish this task requires that we gather information on genetic translation rates in a wide diversity of different organisms in the biosphere, another area that lacks information.

In summary, the information, or DNA, approach to understanding the biosphere offers a new way to approach a quantitative analysis of the biosphere that impinges on a number of areas of environmental and biological sciences, including evolutionary biology, biomass production rates, and biological computational capacity. This approach may help us understand the changing complexity of the biosphere over time and to predict in new ways, both anthropogenic and natural, future changes in the biosphere.

## Supporting Information

S1 Fig Calculating total DNA. The calculation method used to work out the DNA quantity (Mb) per major group of organisms using either organism density or number data from the literature, as described in detail here.
(TIF)

S2 Fig Distribution of genome sizes used in calculations. Values used in calculations are highlighted in red. a) Prokaryote genome sizes extracted by the PFGE method. b) Distribution of genome sizes extracted by complete sequencing. c) Distribution of plant genome sizes. d) Distribution of animal genome sizes. e) Distribution of viral genome sizes. f) Distribution of algal genome sizes. g) Distribution of protist genome sizes. h) Distribution of fungal genome sizes.
(TIF)

S1 Methods Methods of calculating total DNA with errors and uncertainties. (DOCX)

S1 Table Animal number counts and biomass estimates. The total weights of termites, fish, and krill were calculated using density estimates; hence values of the numbers of individuals and mass of individuals are not included.
(XLSX)

S2 Table Area and animal biomass density applied to different biomes. (XLSX)

S3 Table Including all uncertainties. The uncertainty for the quartiles was achieved by taking the difference between each quartile and the mean, represented here. The standard deviation listed is the standard deviation of a smaller, symmetric range around the mean. The exception to these uncertainties is prokaryotes, where the first and second uncertainties correspond to the Kallmeyer et al. and Whitman et al. ranges, respectively.
(XLSX)
