# Supplementary material for: An Estimate of the Total DNA in the Biosphere
Source: PLoS Biol. 2015 Jun 11;13(6):e1002168. doi: 10.1371/journal.pbio.1002168 (PMC4466264; doi:10.1371/journal.pbio.1002168)
Supplement: S1 Methods — (DOCX) [file pbio.1002168.s003.docx]

# S1 Methods.

**Methods of calculating total DNA**

For all data, one or several conversions were needed to deduce a final estimate of the total DNA content. Typically, the data on organism abundance was given in the literature as carbon content (most frequently in units of Pg of C (petagram of carbon), number of cells, or biomass, most frequently in units of Gt (gigatonne)). All biomass estimates were converted into a total number of cells. From here, the genome size, which is the quantity of DNA per cell, given in megabases (Mb) or picogram (pg), was used to find the total amount of DNA within the group (S1 Fig.).

To estimate the total information content of the biosphere, DNA was quantified in five major subgroups of life: prokaryotes, plants, animals, unicellular eukaryotes (sometimes referred to as protists) and fungi. For each group, available quantifications of biomass, number of individuals, or their respective densities were converted to DNA quantities through appropriate conversions including average genome size.

For prokaryotes, there were two main estimations of abundance, 9.2 – 31.7 x 10^29^ cells by Kallmeyer *et al.* [1] and 4 - 6 x 10^30^ cells by Whitman *et al.* [2]. Both sources give their estimate as a range, hence the midpoint of the range was chosen for further calculations. The estimations differ by an order of magnitude. For the purposes of this work, Whitman's value with midpoint 5 x 10^30^ cells was used, as an additional paper by McMahon and Parnell independently derives a deep continental subsurface microbial biomass of 14-135 Pg of C, which overlaps the Whitman estimate of 22-215 Pg of C [3]. The prokaryote genome size was calculated from the mean derived from a table by Gregory [4]. This gave the average genome size as 3.2147 Mb. For prokaryotes, the estimated total number of cells was combined with the average prokaryotic genome size to give the total amount of DNA contained in the group.­­

For plants, the average biomass from four different estimates, 561.8 Gt of carbon [2] 520 Gt of carbon [5], 1841 Gt biomass [6], and 890 Gt biomass [7] was converted to the number of cells assuming carbon content is 50% of dry weight and using a plant cell mass of 2 x 10^-10^ g [8] by assuming that plant cells are an order of magnitude larger in each dimension than a prokaryotic cell and lastly converted to a total amount of DNA of 3.65 x 10^31^ Mb. The genome size used was derived from data from the Kew Gardens Plant DNA C-values Database, by taking the mean of all entries in the database giving an average genome size of 5958.01 Mb [9]. The mean from the four estimates was used to estimate the total DNA content of plants.

DNA quantities in animals were found using two methods. The first method used an estimate for the total biomass in major subgroups of animals, mostly mammals, insects and fish (S1 Table 1).

These values were converted to a total number of cells using a eukaryotic cell mass of 10^-9^ g [10]. For each group, the number of cells was combined with the average genome size for that group by taking the mean of the relevant available genome size entries in the Animal Genome Size Database [11], before the total DNA amount was summed from the individual contributions, to give a final DNA quantity in animals of 4.24 x 10^29^ Mb.

Most animals are diploid, meaning that the total amount of DNA per cell is actually twice the genome size. Here, the genome size of animals was multiplied by a factor of two to get the total DNA content per cell and thus for the whole group. Plants and fungi typically vary between being haploid and diploid at different stages of their life. Prokaryotes have between 1 and 4 copies of their genome in every cell, but the number of copies can be much greater. These variations do not alter the order of magnitude estimate of total DNA. Here we only take into account the diploid characteristics of DNA for animals.

Another way to estimate the total quantity of DNA in animals was to combine data of animal biomass density from various sources. Several detailed case studies investigating the density of animals within a limited area were conducted in the 1960s-1980s. These typically totalled the final biomass of animals within the studied area, giving a final estimate of animal biomass density. The surveys of animal biomass density used here were a Belgian oak- hornbeam forest [12,13], Nairobi National Park [14,15], Gabon lowland rainforest [16], Serengeti [15,16], Venezuelan Amazon [16,17], Ontario lakes [18], New Zealand forest [13], Brazilian ranch [19], Zaire-Congo deciduous woodland [13,20], Central Amazonian rainforest [21], Puerto Rico montane rainforest [13,22], India tropical evergreen forest [16,23] and Borneo [16,24].

In order to apply these estimates to all biomes, biomass estimates were also constructed for deserts, tundra and the poles. For deserts, the average density of animals was taken to be 27.5 individuals/ha, which is the midpoint of 14-41 individuals/ha [25]. The animals inhabiting this region were taken to be jack rabbits, kangaroo rats, kangaroo mice, pocket mice, grasshopper mice, and antelope ground squirrels [25], and using their average mass per individual gave a total biomass in this regime of 8333 kg/km^2^. For the tundra, an average biomass of 50 kg/km^2^ was chosen from several estimates of total and ungulate biomass in tundra in Siberia and Canada [26-28]. Lastly, the estimate of biomass on the poles was made by assuming that the biomass consists mainly of penguins, more specifically 80% of the biomass [29], and calculating the penguin biomass density in Antarctica. The total number of breeding pairs of penguins in Antarctica is estimated to be 20 million [30]. The average weight of a penguin was taken to be 14 kg. Taken together, this generated a total penguin biomass of about 5.7 x 10^7^ kg. Letting this account for 80% of the Antarctic biomass and dividing by the area of Antarctica, approximately 1.4 x 10^7^ km^2^ [31], a biomass density of 5.165 kg/km^2^ was achieved. This was then used to represent the polar biomass density. These biomass densities for different biomes were then multiplied by the area of different biomes on Earth. The biome data was retrieved from The Conservation Gateway of The Nature Conservancy [32,33]. S1 Table 2 shows the ecoregional data used, together with the biomass density applied to each region, and finally the total biomass in each. The total mass within each biome was then combined with the total mass of four groups from the previous section which did not enter into this calculation, namely humans, krill, marine fish and whales. This total mass was then divided by the mass per cell, which was taken to be the mass of a human cell, 10^-9^ g/cell [10], in order to get an estimate of the total number of cells. The total number of cells was then combined with the animal average genome size of 4456 Mb [11]. This generated a total DNA content in animals of 3.67 x 10^29^ Mb.

Few quantifications of protists were found. Extensive literature reviews indicate that there is no overall estimate of abundance of individual protists or protist biomass on Earth. Hence, case studies of the density of protists in certain areas were used to reach an estimate of the total DNA. This was split up firstly into two groups: algae and other protists. For algae, a density in water of 10^2^ cells/ml was used [34]. To obtain a total number of algal cells, the total volume of aquatic habitats containing prokaryotes from Table 1 in Whitman *et al.* [2] was used, on the assumption that algal and prokaryote habitats are essentially the same. This gave a total number of algae of 1.37 x 10^26^ cells. The mean algal genome size was retrieved from the Kew Gardens Algal C-value Database [35], and was found to be 855.89 Mb. Multiplying the total number of algal cells by the mean genome size gave a total amount of DNA in algae of 1.17 x 10^29^ Mb.

Next, terrestrial protists were assessed using a range of estimates for mainly the density of certain subgroups such as ciliates, amoebae and testacea. A number of density counts exist, ranging between 60-349,500 individuals/g of soil. These estimates come from a variety of countries and biomes, more specifically Austria [36], Australia [37], Puerto Rico [38], Scotland [39] and the US [40,41]. The mean density from these sources was taken, giving a protist density of 212,345 cells/g soil. In order to quantify the amount of soil on Earth, the amount of soil used by Whitman et al [2] was taken from Table 2 by multiplying all ecosystem areas by a depth of 8 m, apart from tundra, boreal forest and alpine soils, which are 1m deep [2]. This method gave a total amount of soil of 8.4 x 10^17^ litres. The density of soil of 1.3 x 10^6^ g/m^3^ gave a total mass of soil on Earth of 1.092 x 10^21^ g [2]. The total number of the protists on Earth is thus 2.32 x 10^26^ cells. The average protist genome size is 59.529 Mb [42]. Using this, the total amount of DNA in land protists was found to be 1.38 x 10^28^ Mb. Finally, the DNA amounts of land protists and algae were combined. This gave a total protistan DNA quantity of 1.31 x 10^29^ Mb.

A similar method used to determine the density counts of animals and protists was used for estimating the total fungal biomass. Three estimates of fungal density in forests were used: 24 kg/ha in a Yukon boreal forest [43], 29.4 kg/ha in a Pyrenean pine forest [44] and 8.8 kg/ha from a Swedish spruce forest [45]. The average of these estimates was multiplied by the total area of the sum of forest biomes on the list from The Nature Conservancy [32,33] resulting in a global fungal biomass of 1.25 x 10^11^ kg. Using the same eukaryotic cell mass of 2 x 10^-10^ g/cell as calculated earlier gave a total cell count of 6.23 x 10^23^ cells. Multiplying this by the average fungal genome size of 31.874 Mb [46] resulted in a DNA quantity of 1.99 x 10^25^ Mb. However, the majority of fungi are found below ground, hence, dividing the above-ground DNA amount by 0.0115 [47] raised the total DNA quantity in fungi to 1.73 x 10^27^ Mb.

Viruses also contribute to the total DNA available on Earth. The total number of viruses on Earth has been estimated at 10^31^ [48,49], which combined with an average viral genome size of 0.039518 Mb from the Entrez Genome database for viruses [50] gives a DNA content in viruses of 3.95 x 10^29^ Mb.

The combined values above give a total DNA amount in the biosphere of 5.35 x 10^31^ (±3.55 x 10^31^) Mb.

**Errors and uncertainties**

According to the nature of the methods here, it is important to consider error ranges and uncertainties which are propagated within the calculations. Quantifying errors and ranking them according to their relative effect on the total DNA amount will help identify the areas that are in most urgent need of improvement in order to enhance the accuracy of the calculations that we present. Not all values that went into these calculations had a quantified error or uncertainty explicitly associated with them as presented in the literature, but all groups of organisms had an uncertainty quoted for at least one value that went into the calculations. Hence, all existing uncertainties were propagated during the calculations presented to obtain the total DNA quantity for each group. When more than one value had an uncertainty, the uncertainties were combined according to standard methods of error combination, to give a total error for each group. Lastly, the errors from each group were combined in order to get a final uncertainty in the total estimate of DNA quantity on Earth.

A source of uncertainty common to all groups was the distribution of genome sizes for that specific group. In all calculations for the total quantity of DNA, the mean of the genome size from a database was used. However, the genome size distributions is not Gaussian in shape, but rather very skewed towards the lower genome sizes. Hence, for every group, the genome size distribution and its uncertainties were analysed as described below.

**Uncertainties for prokaryotes**

Prokaryotes were somewhat of an anomalous group in that there are two different databases with genome sizes, which have been extracted by different methods. The genome size database chosen for the total value of DNA was achieved through the pulse-field gel electrophoresis (PFGE) method [4], and was chosen for consistency as it is an offshoot from

the animal genome size database, which was used for the animal calculations [11]. This

database contains 681 entries, and the genome size distribution can be seen in S2 Fig.

As evident from S2 Fig., the genome size distribution has a positive skew towards the lower genome sizes, with skewness = 1.10 and kurtosis = 0.884. These values are very low compared to those of other distributions. Hence, it was determined that this distribution was close enough to a Gaussian distribution to be able to use the standard deviation as the uncertainty range, with *σ* = 2.0 Mb. The peak of the distribution was identified to lie in the 1.8 Mb bin, which is within one standard deviation of the mean *μ* = 3.215. The standard deviation *σ* = 2.0 Mb was hence the uncertainty in the genome size that was used for the calculations on the total uncertainty for prokaryotes.

For completeness, the distribution of genome sizes from another database, the Center for Biological Studies Genome Atlas Database, was included to exhibit the similarities [51]. This distribution can be seen in S2 Fig., and has 1140 entries, a mean of *μ* = 3.535, standard deviation of *σ* = 1.932, skewness = 0.806 and kurtosis = 0.789. The peak can be seen to lie in the 1.9 Mb bin.

The other source of uncertainty in the prokaryote calculations was taken from the initial range with which the number of cells were quoted. Whitman quotes total cells in a range of 4 - 6 x 10^30^ cells [2], whilst Kallmeyer et al quotes a range of 9.2 – 31.7 x 10^29^ cells [1], corresponding to ranges of 2 x 10^30^ cells and 2.25 x 10^30^ cells respectively. The larger of these was divided in half and combined with the uncertainty in genome size to give a final uncertainty of 1.06 x 10^31^ Mb for the total DNA amount in prokaryotes.

**Uncertainties for plants**

The genome size distribution for plants is seen in S2 Fig. The Kew Gardens Plant C-values Database on which the distributions are based contained a total of 8509 entries [9].

The data show that plant genome sizes are heavily skewed towards low genome sizes, with skewness = 3.902 and kurtosis = 25.65. One way to quantify the uncertainty of this distribution was to find the quartiles, with Q1 = 817 Mb and Q3 = 6944 Mb. An uncertainty could then be stated as the difference between each quartile and the mean, *μ* = 5958 Mb. Another way to quantify the uncertainty was accomplished by assuming that the distribution around the peak was roughly Gaussian, and calculating the standard deviation of a symmetric range around the peak. With the peak in the 1200 Mb bin, the standard deviation of all values up to the 2400 Mb bin was found to be *σ* = 278 Mb. Of the three quantifications, Q1 gave the largest uncertainty, and was used to find the total uncertainty for plants.

The other uncertainty in the plant calculations came from the distribution of estimates of total biomass in plants. Four estimates of total biomass existed, and a Gaussian distribution was assumed, hence the standard deviation *σ* = 2.11 x 10^27^ cells was used. Combining this uncertainty with that of Q1 from the genome size gave a total uncertainty in plant DNA of 3.39 x 10^31^ Mb.

**Uncertainties for animals**

*Uncertainties in biomass by major subgroups.* In the calculations of total animal DNA quantity by adding up major subgroups, there were often uncertainties quoted in the number of individuals, the average weight of an individual or the total biomass of a subgroup. Another source of uncertainty lay in the genome size for each subgroup, where there was a spread of values for the genome size even within the same species. Hence, the standard deviation of the genome size distribution for each subgroup was used to quantify an uncertainty. For each subgroup, these uncertainties were propagated through the calculations in the same way as described earlier and the uncertainties were combined as necessary. The total uncertainty for the method of summing animal biomass of major subgroups was found to be 1.52 x10^29^ Mb when combined according to a summed total.

*Uncertainty for biomass by density by biome.* For this method of calculating total animal DNA quantity using density in different biomes, the genome size distribution for animals as a whole is relevant. This distribution is based on 6517 entries in the Animal Genome Size Database [11]. In the calculations of total DNA quantity, the mean of the genome size distribution was used. However, like the genome size distributions for plants and prokaryotes, animal genome size also exhibit a decidedly positive skew towards the low genome sizes, as seen in S2 Fig.

This distribution has a mean of *μ* = 4456.7 Mb, skewness = 5.257 and kurtosis = 36.87. To quantify the spread, the quartiles were found to be Q1 = 909 Mb and Q3 = 3227 Mb. The peak value in the distribution was in the 1180 Mb bin, hence the standard deviation for all values up to the 2360 Mb bin was found, which gave a value of *σ* = 563.588 Mb. However, the difference between the mean and Q1 gave a larger uncertainty than the standard deviation, hence this was used to calculate the final uncertainty in animal DNA quantity.

The raw data used for the estimate of animal DNA quantity by density was in the form of case study measurements of biomass per area. However, none of the original case studies quoted an uncertainty with their results. Therefore, an uncertainty was quantified by assuming that one can consider the animal biomass to be fairly uniform in most temperate, forested areas. By making this assumption, and excluding any obvious outlier values, a distribution of the biomass density estimates can be found. The quartiles were found to be Q1 = 1125 kg/km^2^ and Q3 = 9700 kg/km^2^, giving an interquartile range of ΔQ = 8575 kg/km^2^, which was used as the uncertainty for the biomass density. The calculations for total DNA quantity in animals was carried through as described earlier, and the uncertainties in the biomass density and genome size combined to give a total uncertainty in animal DNA content of 3.97 x 10^29^ Mb.

**Uncertainties for viruses**

For viruses, the only quantifiable uncertainty comes from the distribution of genome sizes. The database from Entrez Genome contained 3843 entries [50]. The distribution can be seen in S2 Fig. The viral genome size has a mean of *μ* = 0.03952 Mb. Again, it is a highly skewed distribution with skewness = 12.36 and kurtosis = 254.2, and quartiles Q1 = 0.005413 Mb and Q3 = 0.043016 Mb. Again, the standard deviation was taken around the peak of the distribution, up to 0.02 Mb, as there was a distinct cut off at that point, yielding *σ* = 0.043016 Mb. The difference between the first quartile Q1 and the mean gave the largest uncertainty range, hence this was used to calculate the overall uncertainty. By carrying this uncertainty through the calculations described earlier a total uncertainty of 3.50 x 10^29^ Mb for the DNA amount in viruses was achieved.

**Uncertainties for protists**

Separate calculations of uncertainties were conducted for algae and land protists before being combined to give an overall uncertainty in the DNA quantity in protists. For algae, the quantifiable uncertainty exists in the algal genome size, which was acquired from the Kew Gardens Algal C value Database [35] (S2 Fig.).

The algal genome size distribution had 253 entries and displays the trend of being positively skewed towards low genome sizes, with skewness = 6.906 and kurtosis = 53.30. The quartiles were found to be Q1 = 195.6 Mb and Q3 = 684.6 Mb. A standard deviation was taken for a subset of the distribution around the peak of 200 Mb, giving *σ* = 96.07 Mb. The first quartile gave the largest deviation from the mean, hence this difference was used as the uncertainty for algal genome sizes. By carrying this uncertainty through in the calculations, a total uncertainty in the DNA quantity in algae of 9.06 x 10^28^ Mb was found.

For land protists, there are uncertainties attached to both the genome size and the protist density in soil. The genome size distribution came from the Ensembl Protist Database with 23 entries [42]. Again, even with significantly fewer entries in this database compared to other groups, the protist genome size distribution also shows a positive skew towards lower genome sizes, with skewness = 1.83 and kurtosis=3.62. The peak lies in the 30 Mb bin, so an uncertainty range of about 20 Mb was assumed. The quartiles were assessed to be Q1 = 23.26 Mb and Q3 = 84.13 Mb. The first quartile has the largest deviation from the mean, hence the difference between the mean and Q1 was used as the uncertainty in genome size.

Furthermore, by expecting the protist density in soil to be normally distributed, the standard deviation of these measurements was found to be *σ* = 398,649 protists/g of soil. This uncertainty was propagated through the calculations and combined with the uncertainty in genome size, giving a final uncertainty in DNA quantity found in land protists as 2.7 x 10^28^ Mb. Lastly, the DNA quantities found in algae and protists were added together, and the uncertainties combined to give an overall protistan DNA quantity uncertainty of 9.46 x 10^28^ Mb. In order to understand the reliability of the estimate of the total amount of DNA in protists, a separate calculation of total DNA amounts was conducted, using a different reference as a starting point. One estimate is that there are roughly 5 x 10^6^ protists per teaspoon of soil [52]. This gives an average protist density in soil of 10^9^ protists per litre soil. The total volume of soil on Earth is 8.4 x 10^17^ litres [2]. This gives a total number of protists in soil of 8.4 x 10^26^ protists. The mean genome size of protists was taken as 59.529 Mb [42]. Hence, this gives a total DNA amount in protists of 5 x 10^28^ Mb. This gives a total quantity of DNA in protists which is within one order of magnitude of the result from the previous method, lending credibility to the size of the protist estimate.

**Uncertainties for fungi**

The calculations for the total fungal DNA amount included uncertainties in biomass density and genome size. The biomass density uncertainties were quoted in the original research, and propagated through the calculations. The fungal genome size database had 1590 entries [46] and the genome size distribution is illustrated in S2 Fig.

Like the other genome size distributions, there is a positive skew towards the lower genome sizes, with a mean *μ* = 31.874 Mb, skew = 12.95, kurtosis = 210.88 and quartiles Q1 = 19.3 Mb and Q3 = 36.4 Mb. Notably, in this distribution, the mean value corresponds with the most populated bin at 30 Mb. The standard deviation was calculated for all genome sizes up to 60 Mb, giving *σ* = 10.72 Mb. The largest difference from the mean was found for Q1, hence this was used as the uncertainty in genome size. This was combined with the uncertainty in biomass density and carried through the calculations to give a total DNA quantity uncertainty of 3.37 x 10^27^ Mb in fungi.

As for protists, an alternative derivation of the DNA amount in fungi exists. The density of fungi is 186 μg fungi per g organic matter, by taking the midpoint of the estimate of 155-217 μg fungi per g organic matter [47]. The total amount of organic carbon is 1.477 x 10^18^ g [53], which, if assuming carbon content is 50% of the overall mass, gives a total mass of organic matter on Earth as 2.954 x 10^18^ g. Hence, the total mass of fungi was found to be 5.494 x 10^14^ g. The genome size was taken as 31.874 Mb [46] and the weight of a fungal cell to be the same as a plant cell, 2 x 10^-10^ g, giving a total DNA content for fungi in soil of 8.75 x 10^25^ Mb. This estimate is two orders of magnitude lower from the previous. For our calculations, we assumed the larger of the two values. However, neither value influences the total order of magnitude DNA quantity calculated, which is dominated by prokaryotes and plants in these calculations.

S1 Table 3 shows all of the uncertainties calculated for the different groups. Since most of the uncertainties had a component of the genome size distribution uncertainties, they are listed in these three categories of the first quartile, third quartile and standard deviation.

As described, the uncertainty for the quartiles was achieved by taking the difference between each quartile and the mean. The standard deviation listed is the standard deviation of a smaller, symmetric range around the mean. The exception to these uncertainties is prokaryotes, where the first uncertainty corresponds to using the Whitman et al. range [2], and the second to the Kallmeyer et al. range [1]. By combining the biggest uncertainties from the different groups, the total uncertainty in the amount of DNA on Earth was found to be 3.55 x 10^31^ Mb. Hence, the total estimate of DNA on Earth can be quoted as 5.3 (3.6) x 10^31^ Mb.

**References**

1. Kallmeyer J, Pockalny R, Adhikari RR, Smith DC, D’Hondt S. Global distribution of microbial abundance and biomass in subseafloor sediment. Proc Natl Acad Sci U S A. 2012;109(40): 16213-16216. doi:10.1073/pnas.1203849109.

2. Whitman WB, Coleman DC, Wiebe WJ. Prokaryotes: The unseen majority. Proc Natl Acad Sci USA. 1998;95: 6578-6583.

3. McMahon S, Parnell J. Weighing the deep continental biosphere. FEMS Microbiol Ecol. 2014;87(1): 113-120.

4. Gregory TR. Prokaryote genome sizes Table 1; 2005. Database: genomesize [Internet]. Accessed: http://www.genomesize.com/prokaryotes/table1/.

5. Wang YP, Law RM, Pak B. A global model of carbon, nitrogen and phosphorus cycles for the terrestrial biosphere. Biogeosci Discussions. 2009;6: 9891-9944.

6. Duchesne LC, Larson DW. Cellulose and the evolution of plant life. BioScience 1989;39: 238-241.

7. Rodell M, Chao BF, Au AY, Kimball JS, McDonald KC. Global biomass variation and its geodynamic effects: 1982-98. Earth Interact. 2005;9: 1-19.

8. Adapted from prokaryotic cell mass of 2 x 10^-13^ g and assuming eukaryotic cells are 10 times larger by length, from Whitman WB, Coleman DC, Wiebe WJ. Prokaryotes: The Unseen Majority. P. Natl. Acad. Sci. USA. 1998;95: 6578-6583.

9. Bennett MD, Leitch IJ. Plant DNA C-values database (Release 6.0, Dec. 2012); 2012. Database: data.kew [Internet]. Accessed: http://data.kew.org/cvalues/.

10. Zimmer C. How Many Cells Are In Your Body? 2013 October 23 [cited 23 April 2015]. In: Phenomena: The Loom [Internet]. National Geographic Society. Available: http://phenomena.nationalgeographic.com/2013/10/23/how-many-cells-are-in-your-body/.

11. Gregory TR. Animal genome size database; 2014. Database: genomesize [Internet]. Accessed: http://www.genomesize.com/.

12. Duvigneaud P. La synthese ecologique. Paris: DOIN; 1980.

13. Brockie RE, Moeed A. Animal biomass in a New Zealand forest compared with other parts of the world. Oecologia. 1986;70(1): 24-34.

14. Foster JB, Coe MJ. The biomass of game animals in Nairobi National Park, 1960-66. J Zool. 1968;155(4): 413-425.

15. Delany J, Happold DCD. Ecology of African mammals. Tropical ecology series. United Kingdom: Longman; 1979.

16. Prins HHT, Reitsma JM. Mammalian biomass in an African equatorial rain forest. J Anim Ecol. 1989;58(3): 851-861.

17. Terborgh J. Five New World Primates: A Study in Comparative Ecology (Monographs in Behaviour and Ecology). Princeton: Princeton University Press; 1983.

18. Smokorowski KE, Kelso JRM. Trends in fish community structure, biomass, and production in Three Algoma, Ontario, Lakes. Water, Air and Soil Pollution: Focus. 2002;2(1): 129-150.

19. Schaller GB. Mammals and their biomass on a Brazilian ranch. Arquivos de Zoologia. 1983;31(1): 1-36.

20. Malaisse F, Alexandre J, Freson R, Goffinet G, Malaisse-Mousset M. The miombo ecosystem: a preliminary study. In: Golley PM, Golley FB, editors. Tropical ecology with an emphasis on organic production. Athens: University of Georgia Press; 1972. p 418.

21. Fittkau EJ, Klinge H. On biomass and trophic structure of the Central Amazonian rain forest ecosystem. Biotropica. 1973;5(1): 2-14.

22. Odum HT, Abbott W, Selander RK, Golley FB, Wilson RF. Estimates of chlorophyll and biomass of the tabanuco forest of Puerto Rico. In: Odum HT, Pigeon RF, editors. A tropical rain forest. U.S. Atomic Energy Commission; 1970. pp 3-19.

23. Balakrishnan M. The larger mammals and their endangered habitats in the Silent Valley Forests of South India. Biol Conserv. 1984;29(3): 277-286.

24. Wilson WL, Johns AD. Diversity and abundance of selected animal species in undisturbed forest, selectively logged forest and plantations in East Kalimantan, Indonesia. Biol Conserv. 1982;24(3): 205-218.

25. The desert biome. The Biomes Group UC Berkeley. 2006. Available: http://www.ucmp.berkeley.edu/glossary/ gloss5/biome/deserts.html.

26. Krause H. Chapter 11: Plant production and prey biomass. 2005. Available: http://www.hanskrause.de/HKHPE/hkhpe_53_11.htm.

27. Hudson RJ, Bunnell FL. Grazing in Tundra and Northern Boreal Environments. In: Morley FHW, editor. World Animal Science: Grazing Animals. Amsterdam: Elsevier; 1980. pp. 203-223.

28. Redmann RE. Production and diversity in contemporary grasslands. In: Hopkins DM, Matthews JV, Schweger CE, Young SB, editors. Paleoecology of Beringia. USA: Academic Press; 1982. pp. 223-239.

29. Biodiversity in Antarctica and the Southern Ocean. WWF. 2014. Available: http://www.wwf.org.au/our_work/saving_the_natural_world/oceans_and_marine/priority_ocean_places/antarctica_and_southern_ocean/biodiversity/.

30. Penguins. British Antarctic Survey. 2014. Available: http://www.antarctica.ac.uk/about_antarctica/wildlife/birds/penguins/.

31. Sizing up Antarctica. Discovering Antarctica. 2014. Available: http://www.discoveringantarctica.org.uk/multimedia/flash/2_sizingup.html.

32. Ecoregional data. The Nature Conservancy. 2014. Available: http://www.conservationgateway.org/ConservationPlanning/ToolsData/CoreData/EcoregionalData/Pages/ecoregional-data.Aspx.

33. Pasquarella V. Location and area of biomes. 2014. Available: http://gk12glacier.bu.edu/wordpress/pasquarella/data-sets/location-and-area-of-biomes/.

34. Rahman MM, Jewel MAS, Khan S, Haque MM. Study of euglenophytes bloom and its impact on fish growth in Bangladesh. Algae 2007;22: 185-192.

35. Kapraun DF, Leitch IJ, Bennett MD. Algal DNA C-value database (Release 1.0, Dec. 2004); 2004. Database: data.kew [Internet]. Accessed: http://data.kew.org/cvalues/.

36. Berthold A, Palzenberger M. Comparison between direct counts of active soil ciliates (Protozoa) and most probable number estimates obtained by Singh's dilution culture method. Biol Fert Soil. 1995;19: 348-356.

37. Robinson BS, Bamforth SS, Dobson PJ. Density and diversity of protozoa in some arid Australian soils. J Eukaryot Microbiol. 2002;49(6): 449-453.

38. Bamforth SS. Protozoa from aboveground and ground soils of a tropical rain forest in Puerto Rico. Pedobiologia. 2007;50: 515-525.

39. Esteban GF, Clarke KJ, Olmo JL, Finlay BJ. Soil protozoa - an intensive study of population dynamics and community structure in an upland grassland. Appl Soil Ecol. 2006;33: 137-151.

40. Bamforth SS. Distribution of and insights from soil protozoa of the Olympic coniferous rain forest. Pedobiologia. 2010;53: 361-367.

41. Bamforth SS. Protozoa of biological soil crusts of a cool desert in Utah. Journ Arid Environ. 2008;72: 722-729.

42. European Bioinformatics Institute (2013) Ensembl protists species; 2013. Database: ensembl [Internet]. Accessed: http://protists.ensembl.org/info/about/species.html.

43. Krebs CJ, Carrier P, Boutin S, Boonstra R, Hofer E. Mushroom crops in relation to weather in the southwestern Yukon. Botany. 2008;86(12): 1497-1502.

44. Martínez de Aragón J, Bonet JA, Fischer CR, Colinas C. Productivity of ectomycorrhizal and selected edible saprotrophic fungi in pine forests of the pre-Pyrenees mountains, Spain: Predictive equations for forest management of mycological resources. For Ecol Manage. 2007;252(1-3): 239-256.

45. Dahlberg A, Jonsson L, Nylund J-E. Species diversity and distribution of biomass above and below ground among ectomycorrhizal fungi in an old-growth Norway spruce forest in South Sweden. Can J Bot. 1997;75(8): 1323-1335.

46. Kullman B, Tamm H, Kullman K. Fungal genome size database; 2005. Database: zbi [Internet]. Accessed: http://www.zbi.ee/fungal-genomesize/.

47. Dighton J, White JF Jr, Oudemans P. The Fungal Community: Its Organization and Role in the Ecosystem. 3rd ed. Boca Raton: CRC Press; 2005.

48. Griffin DW. The quest for extraterrestrial life: what about the viruses? Astrobiology. 2013;13: 774-783.

49. Breitbart M, Rohwer F. Here a virus, there a virus, everywhere the same virus? Trends Microbiol. 2005;13(6): 278-284.

50. National Center for Biotechnology Information. Entrez genome viral genomes, 2014. Database: ncbi.nlm.nih [Internet] Accessed: https://www.ncbi.nlm.nih.gov/genomes/GenomesHome.cgi?taxid=10239.

51. Hallin P, Borini S. CBS Genome Atlas Database; 2013. Database: cbs.dtu.dk [Internet]. Accessed: http://www.cbs.dtu.dk/services/GenomeAtlas/.

52. Biggs A, Hagins WC, Kapicka C. Glencoe Science Biology: The Dynamics of Life. New York: McGraw-Hill/Glencoe; 1991.

53. Buringh P. Organic carbon in soils of the world. In: Woodwell GM, editor. The Role of Terrestrial Vegetation in the Global Carbon Cycle. Measurement by Remote Sensing, SCOPE. New York: John Wiley and Sons Ltd; 1984. pp. 91-109.

54. U.S. and World Population Clock. United States Census Bureau. 2014. Available: https://www.census.gov/popclock/.

55. Brown LR. Plan B updates, World's rangelands deteriorating under mounting pressure. Earth Policy Institute. 2005. Available: http://www.earth-policy.org/plan_b_updates/2002/update6.

56. Dairy facts. Purdue University. 2008. Available: http://www.ansc.purdue.edu/faen/dairy%20facts.html.

57. Claeys MC, Rogers SB. Sheep facts. NC State University. 2003. Available: http://www.cals.ncsu.edu/an_sci/extension/animal/4hyouth/sheep/sheepfacts.htm.

58. The Economist Online. Counting chickens. 2011 Jul 27 [cited 24 April 2015]. In: Graphic Detail [Internet]. Available: http://www.economist.com/blogs/dailychart/2011/07/global-livestock-counts.

59. Pork glossary. U.S. Environmental Protection Agency. 2012. Available: http://www.epa.gov/agriculture/ag101/porkglossary.html.

60. Embery J, Lucaire E. Joan Embery's collection of amazing animal facts. New York: Delacorte Press; 1983.

61. Weightlifting ant is photo winner. BBC News. 2010. Available: http://news.bbc.co.uk/1/hi/8526086.stm.

62. Sanderson MG. Biomass of termites and their emissions of methane and carbon dioxide: A global database. Global Biogeochem Cycles. 1996;10: 543-557.

63. Irigoien X, Klevjer TA, Røstad A, Martinez U, Boyra G, Acuña JL, et al. Large mesopelagic fishes biomass and trophic efficiency in the open ocean. Nature Comm. 2014;5: doi:10.1038/ncomms4271.

64. Atkinson A, Siegel V, Pakhomov EA, Jessopp MJ, Loeb V. A re-appraisal of the total biomass and annual production of Antarctic krill. Deep Sea Research Part I: Ocean Res Papers. 2009;56: 727-740.

65. Jeffery NW. The first genome size estimates for six species of krill (Malacostraca, euphausiidae): large genomes at the north and south poles. Polar Biology. 2012;35: 959-962.

66. Williams CB. The range and pattern of insect abundance. The American Naturalist. 1960;9: 137-151.

67. Numbers of insects (species and individuals). Smithsonian Institution. 2014. Available: http://www.si.edu/Encyclopedia_SI/nmnh/buginfo/bugnos.htm.

68. Gaston KJ, Blackburn TM. How many birds are there? Biodiv Cons. 1997;6: 615-625.
